# Transcription Factor ATF3 Participates in DeltaNp63-Mediated Proliferation of Corneal Epithelial Cells

**DOI:** 10.3390/jpm13040700

**Published:** 2023-04-21

**Authors:** Yi-Jen Hsueh, Yaa-Jyuhn James Meir, Hui-Yi Hsiao, Chao-Min Cheng, Hui-Kang David Ma, Wei-Chi Wu, Hung-Chi Chen

**Affiliations:** 1 Department of Ophthalmology, Chang Gung Memorial Hospital, Linkou Branch, Taoyuan 333, Taiwan; 2 Center for Tissue Engineering, Chang Gung Memorial Hospital, Linkou Branch, Taoyuan 333, Taiwan; 3 Department of Biomedical Sciences, Chang Gung University, Taoyuan 333, Taiwan; 4 Institute of Biomedical Engineering, National Tsing Hua University, Hsinchu 300, Taiwan; 5 Department of Chinese Medicine, Chang Gung University, Taoyuan 333, Taiwan; 6 Department of Medicine, Chang Gung University, Taoyuan 333, Taiwan

**Keywords:** tumor protein p63 ΔN isoform, activating transcription factor 3, cyclin-dependent kinases, corneal epithelial cell, cell proliferation

## Abstract

Understanding the regulatory mechanisms underlying corneal epithelial cell (CEC) proliferation in vitro may provide the means to boost CEC production in cell therapy for ocular disorders. The transcription factor ΔNp63 plays a crucial role in the proliferation of CECs, but the underlying mechanisms is yet to be elucidated. TP63 and ΔNp63 are encoded by the *TP63* gene via alternative promoters. We previously reported that both ΔNp63 and activating transcription factor (ATF3) are substantially expressed in cultured CECs, but the regulatory relationship between ΔNp63 and ATF3 is unknown. In the present study, we found that ΔNp63 increased *ATF3* expression and *ATF3* promoter activity in cultured CECs. The deletion of the p63 binding core site reduced *ATF3* promoter activity. CECs overexpressing *ATF3* exhibited significantly greater proliferation than control CECs. *ATF3* knockdown suppressed the ΔNp63-induced increase in cell proliferation. Overexpression of *ATF3* in CECs significantly elevated protein and mRNA levels of *cyclin D*. The protein levels of keratin 3/14, integrin β1, and involucrin did not differ between *ATF3*-overexpressing CECs, *ATF3*-downregulated CECs, and control cells. In conclusion, our results suggest that ΔNp63 increases CEC proliferation via the ΔNp63/ATF3/CDK pathway.

## 1. Introduction

The corneal epithelium is made up of terminally differentiated keratinocytes that are constantly replaced by newly divided cells derived from corneal epithelial stem cells [1]. Because they are localized in the limbus, corneal epithelial stem cells are also known as limbal stem cells (LSCs). LSCs have a high proliferation potential, infinite self-renewal ability, and are capable of migrating forward to the cornea to promote corneal epithelial cell (CEC) proliferation [1,2]. The *TP63* gene encoded two major protein isoforms, TAp63 and ΔNp63, via distinct promoters; both proteins are critical transcription factors for the regulation of epidermal keratinocyte proliferation and differentiation [3]. LSCs express the transcription factor ΔNp63 [4], which plays an important role in LSC proliferation through the cis-transcriptional regulation of cell cycle modulators during epidermal development [5]. Furthermore, ΔNp63 may act as a master regulator of these cells by controlling the switch between proliferation and differentiation [6]. The signal transducer and activator of transcription 3 (STAT3) upregulates ΔNp63; knockdown of *STAT3* expression downregulates ΔNp63, resulting in the suppression of cell proliferation but the promotion of cell differentiation [6]. Elucidation of the role of ΔNp63 in CEC proliferation may broaden our understanding of the regulatory mechanisms responsible for LSC proliferation.

In our previous study [7], six candidate TFs were identified based on putative TF binding sites in the *ΔNp63* promoter: paired box protein 6 (Pax-6), early growth response protein 1 (EGR1), CCAAT increaser binding protein beta (CEBPB), jun proto-oncogene (JUN), activating transcription factor 3 (ATF3), and AT-rich interaction domain 5B (ARID5B). We found that silencing ATF3 did not alter the expression level of ΔNp63, suggesting that ΔNp63 is not a downstream effector of ATF3, although both ΔNp63 and ATF3 are involved in the regulation of CEC proliferation [7]. ATF3, a member of the ATF/cyclic AMP response element-binding (CREB) family of TFs [8,9], upregulates cyclin-dependent kinases (CDKs; cyclin E and cyclin D) to increase cell proliferation [10,11]. Thus, the ATF3/CDK pathway may participate in the regulation of CEC proliferation.

The clinical application of cell therapy and tissue engineering requires the expansion of cell populations. The use of cultured cells for cell therapy has some disadvantages that should be carefully examined and minimized, such as the loss of cell function caused by excessive differentiation and aging and the risk of canceration [12,13]. The DNA binding site for TF tumor protein p63 is highly homologous to that of p53 (about 60%) [14]; therefore, direct transfection of overexpressed p63 may increase the risk of cross-reaction with p53 and cancer induction. We previously demonstrated that STAT3 is an upstream regulator of ΔNp63 [6]. Hence, it is reasonable to assume that the genetic manipulation of STAT3 may prevent cross-reactivity between TF tumor protein p63 and p53. Furthermore, the identification of downstream effectors responsible for ΔNp63-mediated CEC proliferation may also provide insights into the development of genetic manipulation methods to enhance CEC proliferation in vitro. Such a methodological approach may rule out the possibility of cross-reactivity between p63 and p53 and facilitate the development of reliable cell culture systems and cell therapy.

Both ΔNp63 and ATF3 are substantially expressed in CECs; however, ΔNp63 is not a downstream effector of ATF3 [7]. Whether ΔNp63 is an upstream regulator of ATF3 remains to be investigated. To determine the regulatory relationship between ΔNp63 and ATF3, we hypothesize that ATF3 expression is regulated by ΔNp63 in CECs and that ATF3 participates in ΔNp63-mediated CEC proliferation. In the present study, we observed that the ectopically expressed *ΔNp63* increases the expression of ATF3 in cultured CECs, resulting in the promotion of CEC proliferation. These findings suggest that the ΔNp63/ATF3/CDK signaling pathway plays a crucial role in CEC proliferation.

## 2. Materials and Methods

### 2.1. Cell Cultures

Human tissues were obtained in accordance with the principles of the Declaration of Helsinki, and the study protocol was approved by the Institutional Review Board of Chang Gung Memorial Hospital (IRB no. 201901773B0). Human limbal/peripheral corneal tissue was collected within 6 h after corneal transplantation. Rabbit tissues were obtained from the healthy eyes of 2-month-old New Zealand white rabbits. The protocol was approved by the Institutional Animal Care and Use Committee of Chang Gung Memorial Hospital Animal Center. The rabbit eyelids were sterilized with povidone-iodine, and the limbal/corneal tissue was separated from the superficial limbal/corneal stroma using lamellar keratectomy.

Human and rabbit limbal tissues containing CECs and the corneal stroma were cultured in limbal epithelium growth medium (LEGM)/DMEM/Ham’s F12 supplemented with 20 mM HEPES, 5% FBS, 0.5% DMSO, 2 ng/mL mEGF, and 1 µg/mL bovine insulin. The medium was renewed every 2 days. The CECs were harvested on day 14 and subsequently seeded into new culture dishes in LEGM.

### 2.2. Construction of ATF3 Promoter Luciferase Vectors

The sequences of the human *ATF3* promoter (+12 to −1249, sequence according to GenBank: U37542.1) with and without the region of the p63 binding site (p63 motif: 5′-CCCCCAGGCCTGGGCAGGTT-3′ were obtained using the p63scan algorithm software (Radboud University, Nijmegen, The Netherlands, http://www.ncmls.eu/bioinfo/p63scan/ accessed on 1 May 2012) [15,16] and synthesized and cloned into a pGL3-Basic vector (Promega, Madison, WI, USA). The promoters were inserted in the sense orientation between the KpnI and SacI sites to create *ATF3* promoter luciferase report vectors (wt-HuATF3 and del-HuATF3).

### 2.3. Analysis of ATF3 Promoter Activity

Human and rabbit CECs were co-transfected with the appropriate luciferase reporter and β-galactosidase (β-gal) plasmid for 48 h. After transfection, the cells were washed with ice-cold 1 × PBS, and *ATF3* promoter activity was assayed according to the manufacturer’s instruction (Promega). Luciferase activity was quantified using the tFB12 luminometer (Berthold Detection Systems, Pforzheim, Germany), and the β-gal activity was determined using a Sunrise ELISA reader (Tecan, Salzburg, Austria). Luciferase activity was normalized to β-gal activity before analysis.

### 2.4. Establishment of Expression Vectors and siRNAs

The pCMV-*ATF3* plasmid (SC108959) carrying an *ATF3* (NM_001674) human untagged clone was purchased from OriGene Technologies (Rockville, MD, USA). *ATF3* siRNA (si-*ATF3*) was purchased from Santa Cruz Biotechnology (sc-29757; Santa Cruz, CA, USA). *ΔNp63* siRNA (si-*ΔNp63*) with the sequence 5′-CCAUGAGCUGAGCCGUGAA-3′ [6], which was obtained from MWG-Biotech AG (Ebersberg, Germany).

### 2.5. Transfection with Plasmids or siRNAs

For the human *ATF3* promoter assay, human CECs (1 × 10^6^ cells) were mixed with 1.5 μg of pGL3 and 1.5 μg of *β-gal* plasmids. For the rabbit *ATF3* promoter assay, rabbit CECs (1 × 10^6^ cells) were mixed with 2.5 μg of pGL3 and 2.5 μg of *β-gal* plasmids. For the rabbit *ATF3* overexpression assay, rabbit CECs (1 × 10^6^ cells) were mixed with 6 μg of *ATF3* plasmids. For human si-*ΔNp63* assays, human CECs (1 × 10^6^ cells) were mixed with 2.5 μg of si-*ΔNp63* and 2.5 μg of si-*ATF3*. After the addition of 100 μL of nucleofection solution, each cell/DNA mixture was transferred to a 2 mm electroporation cuvette. After nucleofection, 500 μL of prewarmed medium was added to the cuvette, and the cells were transferred into a 12-well plate containing 2 mL of fresh medium. After being incubated for 16 h, the medium was replaced with fresh medium to remove the dead cells.

### 2.6. Infection with ΔNp63 Adenoviral Vectors

An adenoviral *ΔNp63* overexpression vector (Ad-*ΔNp63*) was constructed as previously reported [17]. A recombinant adenovirus carrying the green fluorescent protein gene-encoding gene (Ad-*GFP*) served as a control vector. The adenoviral particles were titered using a QuickTiter Adenovirus Titer ELISA Kit (Cell Biolabs, Inc., San Diego, CA, USA), and Ad-*GFP* was used to evaluate the efficiency of CEC adenoviral transduction. CECs were infected with Ad-*ΔNp63* or Ad-*GFP* vectors. The viral stock (25 μL; 8 × 10^7^ IFU/mL) was added into 0.5 mL of Opti-MEM, and 2 mL of preincubated Opti-MEM was added followed by a 6 h incubation period.

### 2.7. Quantitative Reverse Transcriptase Polymerase Chain Reaction (qRT-PCR) Assay

qRT-PCR was performed using TOOLS 2X SYBR qPCR Mix (BIOTOOLS, New Taipei City, Taiwan) in an ABI StepOne System (Applied Biosystems, Waltham, MA, USA). The qPCR protocol consisted of 40 amplification cycles and a melt-curve analysis at 60 °C for 1 min. The primer sequences for all genes studied are listed in Supplementary Table S1. The amplification curves and Ct values were analyzed using Step One v2.0 software (Applied Biosystems, Waltham, MA, USA). *GAPDH* served as an internal control. All experiments were performed in triplicate, and the qPCR results were analyzed using the delta-delta Ct method.

### 2.8. Western Blot Assays

Total protein was extracted from cultured cells using Tissue Protein Extraction Reagent supplemented with protease and phosphatase inhibitors (Pierce, Rockford, IL, USA). After protein quantification using Coomassie Brilliant Blue, 10 μg of protein per sample was loaded onto a 10% SDS-PAGE gel. After electrophoresis, the proteins were transferred to a PVDF membrane (Pierce). After 1 h blocking in 5% nonfat milk in PBST at room temperature, the membrane was incubated with primary antibodies overnight at 4 °C, followed by 1 h incubation with secondary antibodies at room temperature. ECL Western Blotting Detection Reagents and an Analysis System Kit (GE Healthcare, Amersham, UK) were used to visualize the proteins. The primary antibodies used in this study included those against ΔNp63, ATF3, cyclin A, cyclin B, cyclin D, cyclin E, p27^Kip1^, p21^Cip1^, keratin 3, keratin 14, integrin β1, involucrin, and GAPDH (Santa Cruz Biotechnology). *Image Lab image acquisition* and analysis software (Bio-Rad Laboratories, Hercules, CA, USA) was used to quantify band intensity. The intensity of the protein of interest was normalized to that of GAPDH for each sample.

### 2.9. Proliferation Assays

Cell proliferation was assessed using cell counting, bromodeoxyuridine (BrdU) labeling, and Ki-67 immunofluorescence. The cells were seeded into 6-well culture plates (3.5 × 10^3^ cells/well), and the medium was renewed on the next day. The cells were harvested and counted using a hemocytometer daily from day 1 to day 4.

The BrdU labeling assay was performed using a Cell Proliferation Kit (GE Healthcare). After the 4-day incubation, cells were labeled with BrdU for 24 h and then fixed with 100% methanol for 15 min. The cells were sequentially incubated with anti-BrdU primary antibodies and DNase I for 1 h and with secondary antibodies (Alexa Fluor 488; Invitrogen, San Diego, CA, USA) for 30 min at room temperature, followed by Hoechst 33,342 staining.

In another experiment, after the 4-day incubation, the cells were fixed with 100% methanol for 15 min. The fixed cells were sequentially incubated with anti-Ki67 primary antibodies (MIB-1; Dako, Glostrup, Denmark) for 1 h and with secondary antibodies (Alexa Fluor 594; Invitrogen) for 1 h at room temperature, followed by Hoechst 33,342 staining. The cells were observed under a confocal laser fluorescence microscope (TCS SP2-MP system; Leica, Wetzlar, Germany).

### 2.10. Microarray Analysis

Total RNA was extracted from the cells using an RNeasy column (Qiagen, Valencia, CA, USA) and RNase-free DNase. Oligo-dT served as the primer for reverse transcription. The cDNA derived from control CECs was labeled with Cy3, while the cDNA of ΔNp63-overexpressing CECs was labeled with Cy5. After purification using a cDNA cleanup spin column (Affymetrix, Santa Clara, CA, USA), the labeled cDNA was dissolved in 50 mL of saline citrate (15 mM sodium citrate in 150 mM NaCl) and then hybridized using a human whole-genome oligo array chip (Agilent Technologies, Palo Alto, CA, USA). Microarray image acquisition and analysis were performed as previously described [7].

### 2.11. Statistical Analysis

Continuous data were analyzed using paired *t*-tests. All statistical analyses were carried out using SAS/STAT version 8 (SAS Institute, Cary, NC, USA). The results are expressed as the mean ± SD from three independent experiments. *p* < 0.05 was considered statistically significant.

## 3. Results

### 3.1. ΔNp63 Increases ATF3 Expression by Regulating ATF3 Promoter Activity in CECs

**Figure 1:**
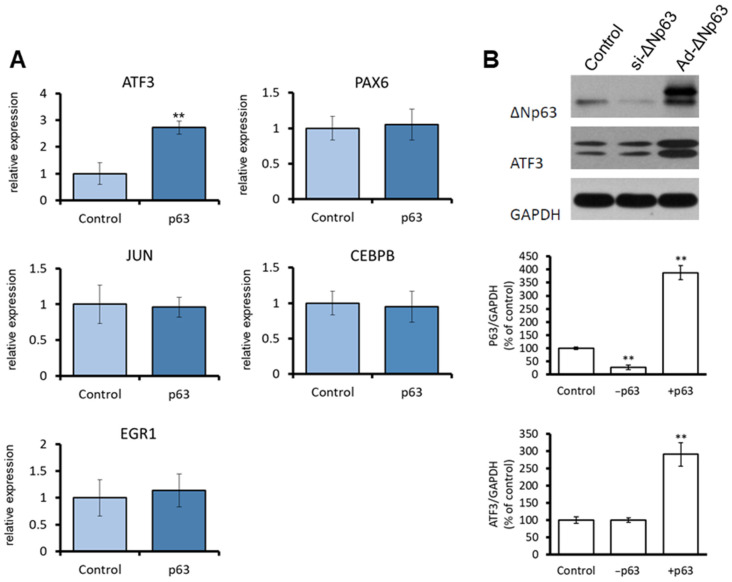
ΔNp63 upregulates ATF3 expression in CECs. (**A**) Human CECs were infected with Ad-*ΔNp63* or Ad-*GFP* (control) for 6 h. Cells were harvested 2 days after infection, and total RNA was extracted to analyze the mRNA expression level of the genes; (**B**) Human CECs were treated with si-*ΔNp63*, Ad-*ΔNp63*, or control plasmids (si-control + Ad-*GFP*). The Cells were harvested 2 days after transfection, and the cell lysates were used to assess the protein levels of ΔNp63 and ATF3 via western blot analysis. ** *p* < 0.01 versus control.

To determine the effects of *ΔNp63* overexpression on CEC proliferation, we carried out microarray analysis in *ΔNp63*-overexpressing human CECs. As shown in Table 1, *ATF3* was upregulated (log_2_ = 3.764) in *ΔNp63*-overexpressing CECs compared to control CECs. As validated by qRT-PCR, overexpression of *ΔNp63* also significantly upregulated *ATF3* expression in CECs (Figure 1A). Western blot results indicated that the protein level of ATF3 was significantly higher in *ΔNp63*-overexpressing (Ad-*ΔNp63*-infected) CECs than that in control CECs (Figure 1B). These findings indicate that ΔNp63 upregulates the mRNA expression of *ATF3* in CECs, resulting in increased ATF3 protein expression.

**Figure 2:**
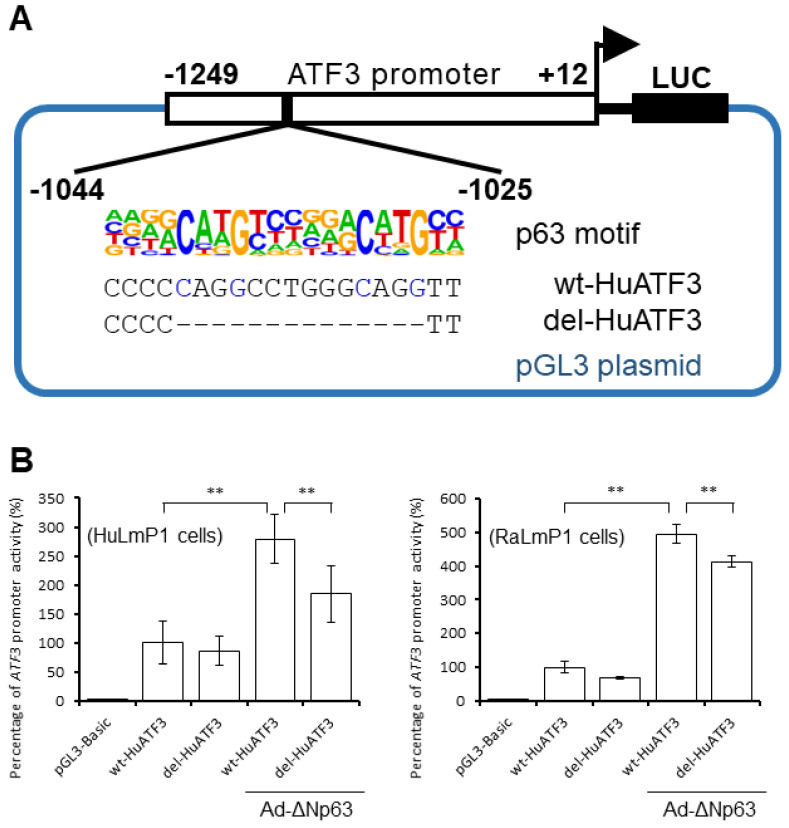
ΔNp63 increases *ATF3* promoter activity in CECs. (**A**) The p63-binding sites in the human *ATF3* promoter region (−1249 to +12) were deduced using p63 motif analysis software (p63scan algorithm). Position weight matrices (matrix sites) were matched to identify p63 motifs (−1044 to −1025); (**B**) Human (HuLmP1) or rabbit (RbLmP1) CECs were co-transfected with *β-gal* plasmids (a transfection control) and either pGL3-Basic, wt-Hu*ATF3*, del-Hu*ATF3*, wt-Hu*ATF3*+Ad-*ΔNp63* or del-Hu*ATF3*+Ad-*ΔNp63* for 16 h. After 48 h of transfection, the cells were harvested and their luciferase activity and β-gal activity were measured. ** *p* < 0.01.

To determine whether ΔNp63 is involved in the regulation of the *ATF3* promotor, p63 motif analysis software (p63scan algorithm) [15] was used to analyze the *ATF3* promoter region (0 to −2000), and a predicted ΔNp63 binding site was identified (Figure 2A). We then examined whether ΔNp63 increases *ATF3* expression via altering *ATF3* promoter activity in CECs. The *ATF3* promoter activity was significantly higher in HuLmP1 cells (human CECs) and RaLmP1 cells (rabbit CECs) co-transfected with wt-Hu*ATF3* and Ad-*ΔNp63* than in those transfected with wt-Hu*ATF3* (pGL3 luciferase reporter vector containing *ATF3* promoter +12 to −1249) (Figure 2B). The *ATF3* promotor activity decreased when the ΔNp63-binding site was deleted (del-Hu*ATF3*) (Figure 2B).

### 3.2. ΔNp63 Promotes CEC Proliferation in an ATF3-Dependent Manner

**Figure 3:**
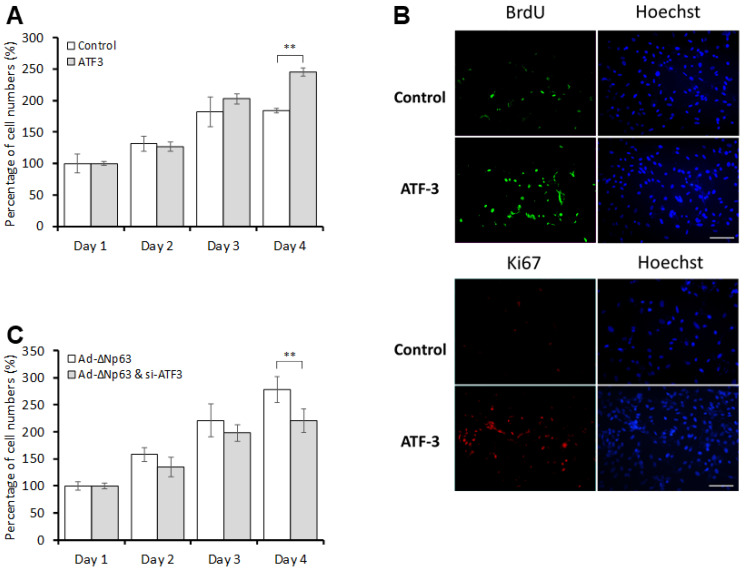
ΔNp63 induces cell proliferation through an ATF3-dependent pathway. (**A**) Rabbit CECs were transfected with pCMV-*ATF3* or control plasmids for 16 h. After transfection, cells were harvested and counted on days 1, 2, 3, and 4; (**B**) Cells were harvested on day 4 and analyzed for proliferation via BrdU assay and Ki-67 staining. Scale bar: 100 μm; (**C**) Ad-*ΔNp63*-infected rabbit CECs were transfected with si-*ATF3* or si-control for 16 h. Cells were harvested and counted on days 1, 2, 3, and 4 after transfection. ** *p* < 0.01.

The extent to which the ΔNp63-mediated induction of *ATF3* expression was associated with cell proliferation in rabbit CECs was investigated using time course experiments. As shown in Figure 3A, there were significantly more *ATF3*-overexpressing CECs than control CECs on day 4. In addition, significantly more BrdU- and Ki-67-positive cells were observed among the *ATF3*-overexpressing CECs compared to the control cells on day 4 (Figure 3B). Knockdown of *ATF3* significantly reduced the number of *ΔNp63*-overexpressing CECs compared to their counterpart cells without ATF3 knockdown on day 4 (Figure 3C). Taken together, *ATF3* expression is critical to CEC proliferation, and *ATF3* knockdown blocks ΔNp63-mediated CEC proliferation.

### 3.3. ATF3 Induces the Expression of Cyclin D in CECs

**Figure 4:**
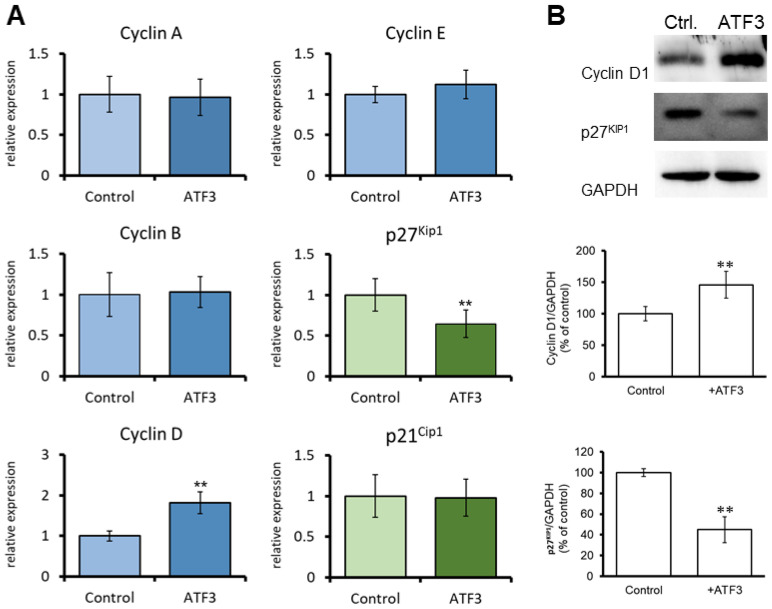
ATF3 regulates the expression of cell-cycle–related genes in CECs. (**A**) Human CECs were transfected with pCMV-*ATF3* or control plasmids for 48 h. After transfection, the cells were harvested, and the expression of cell-cycle–related genes was analyzed by qRT-PCR; (**B**) Human CECs were transfected with pCMV-*ATF3* or control plasmids for 16 h. Cells were harvested on day 4 after transfection and assessed for cyclin D1 and p27^KipP1^ protein expression via western blot analysis. ** *p* < 0.01.

Next, we evaluated the extent to which *ATF3* regulates the expression of cell cycle-related genes, including *cyclin A*, *cyclin B*, *cyclin D*, *cyclin E*, *p27^Kip1^*, and *p21^Cip1^*, in human CECs using qRT-PCR. The results revealed that *ATF3*-overexpressing human CECs exhibited a significantly higher level of *cyclin D* mRNA than control cells (Figure 4A). *ATF3* overexpression did not significantly affect the mRNA levels of *cyclin A*, *cyclin B*, *cyclin E*, *p27^Kip1^*, or *p21^Cip1^.* The cyclin D1 protein level was significantly increased in *ATF3*-overexpressing human CECs compared to that of control cells (Figure 4B). In contrast, the protein level of p27^Kip1^ was significantly decreased in *ATF3*-overexpressing human CECs, although the mRNA level of *p27^Kip1^* was not influenced (Figure 4B).

### 3.4. ATF3 Did Not Affect the Expression of Keratinocyte Differentiation-Related Proteins in CECs

**Figure 5:**
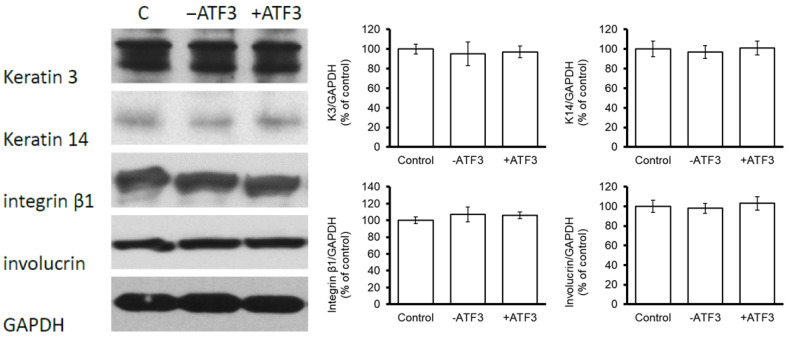
*ATF3* did not alter the expression of keratinocyte differentiation-related proteins in human CECs. Human CECs were transfected with pCMV-*ATF3*, si-*ATF3*, or control plasmids for 16 h. Cells were harvested on day 4 after transfection and assessed for the expression of keratinocyte-differentiation–related proteins via western blot analysis.

The extent to which ΔNp63/ATF3 signaling regulates keratinocyte differentiation in human CECs was then evaluated. As shown in Figure 5, the levels of keratinocyte differentiation-related proteins (keratin 3, keratin 14, integrin β1, and involucrin) did not differ significantly between *ATF3*-overexpressing CECs, *ATF3*-silenced CECs, and control cells. These findings suggest that ATF3 may not participate in the regulation of CEC differentiation.

## 4. Discussion

ΔNp63 was previously shown to be highly expressed in the limbal epithelium and a critical regulator of the differentiation and proliferation of corneal epithelial stem cells [6,18,19]. Our previous study investigated the transcriptional regulation of *ΔNp63* by TF candidates (PAX6, EGR1, CEBPB, JUN, ATF3, and ARID5B) and the roles of those TF candidates in the regulation of CEC proliferation [7]. The present study expands upon these findings and demonstrates that ΔNp63 increased *ATF3* promoter activity, thereby upregulating *ATF3* mRNA expression. ΔNp63 promoted CEC proliferation in an ATF3-dependent manner, and *ATF3* knockdown inhibited ΔNp63-mediated CEC proliferation. ATF3 regulated the expression of the cell-cycle–related proteins cyclin D1 and p27^Kip1^ in CECs, thereby increasing CEC proliferation. However, ATF3 may not be involved in the regulation of CEC differentiation.

Kouwenhoven et al. [15,16] identified the presence of the ΔNp63 DNA-binding profile (5′-NNCNNGNNNNNNCNNGNNN-3′) in a physiologically relevant human cell system and identified several candidate target genes (*DLX5*, *DLX6*, * FOXQ1*, * ZNF143*, * MAFK*, * MAFB*, * SP100*, * HBP1*, * TFAP4*, * ASCL2*, * VDR*, and *RXRA*) and regulatory elements controlled by ΔNp63. Perez et al. [20] found that ΔNp63 binds preferentially to DNA fragments conforming to the 20 bp sequence 5′-RRRC(A/G)(A/T)GYYYRRRC(A/T)(C/T)GYYY -3′. In the present study, we found a ΔNp63 binding site within the *ATF3* promoter (−1043 to −1023) that is involved in the ΔNp63-induced increase in *ATF3* promoter activity. As shown in Figure 2B, we observed that CECs transfected with del-Hu*ATF3* and infected with Ad-*ΔNp63* had higher promoter activity than those transfected with wt-Hu*ATF3* alone. Thus, we hypothesize that ectopically expressed *ΔNp63* may also affect the gene expression of regulators other than *ATF3* to some degree and may indirectly increase the promoter activity of *ATF3* through trans-regulation. Moreover, since our promoter construct covers the *ATF3* promoter ranging from +12 to −1249, the possible involvement of ΔNp63 binding sites further upstream in the *ATF3* promoter (cis regulation) cannot be ruled out. However, because the deletion of the ΔNp63 binding site (−1044 to −1025) significantly decreased the stimulatory effect of ectopically expressed *ΔNp63* on CEC proliferation, we believe that the current findings still support the conclusion that ΔNp63 increases *ATF3* promoter activity.

Overexpression of *ΔNp63* has been shown to downregulate the expression of the cell cycle inhibitor p27^Kip1^ and increase proliferation in human nasopharyngeal carcinoma cells [21]. Furthermore, activated ATF3 elevates the mRNA expression of *cyclin D1* in hepatocytes [22]. However, evidence indicating that ATF3 suppresses the transcription of target genes also has been reported [8]. Thus, the effects of ATF3 on cell physiology vary depending on cell type. For example, ATF3 has been demonstrated to both promote [22,23] and suppress [24,25] cell cycle progression and cell proliferation, as well as mediating both anti- and pro-apoptotic effects [25,26]. The findings of the present study further confirm the relationship between these key regulators during cell proliferation. Our data show that ΔNp63 increased *ATF3* expression by upregulating transcription and that ATF3 regulated the expression of cyclin D and p27 ^Kip1^, resulting in the promotion of CEC proliferation. Notably, both the mRNA and protein levels of *cyclin D* were increased in *ATF3*-overexpressing CECs. However, the protein level of p27 ^Kip1^ was reduced in *ATF3*-overexpressing CECs, while the mRNA level of *p27^Kip1^* was not affected in these cells. These findings imply that ATF3 affects the transcription of *cyclin D* and the protein stability of p27 ^Kip1^ to induce CEC proliferation. Certainly, this possibility merits further investigation. As shown in Figure 3C, the overexpression of *ΔNp63* promoted CEC proliferation by 170% on day 4; however, the lack of an appropriate control group (infected with AD-*GFP*) represents a limitation of this study.

ATF3 downregulates the gene expression of *cyclin A* and *cyclin D1* and activates RUNX2-dependent transcription in maturing chondrocytes [27]. The induction of ATF3 stops cell proliferation and results in the terminal differentiation of chondrocytes [27]. In contrast, the findings of the present study show that *ATF3* overexpression did not affect the expression of the keratinocyte differentiation-related proteins, such as keratin 3, keratin 14, integrin β1, and involucrin, in human CECs, but promoted CEC proliferation. In addition to *ATF3* overexpression/knockdown, the CECs should be treated with or without a commercially available keratinocyte differentiation inducer in order to confirm that there is no effect of *ATF3* overexpression on keratinocyte differentiation in future studies. On the other hand, our previous study showed that STAT3 increases the proliferation of rabbit CECs in a ΔNp63-dependent manner [6]. Inhibition of the ΔNp63 signaling pathway was shown to reduce cell proliferation; however, cell differentiation was concomitantly increased via the repression of integrin β1 expression and promotion of involucrin expression [6]. Given these findings, we suggest that STAT3 may act upstream of ΔNp63/ATF3/CDK signaling to induce CEC proliferation and that suppression of this signaling pathway also increases keratinocyte differentiation.

ΔNp63 plays an important role in LSC stem cell function and the regulation of CEC proliferation [4,6]. Our previous studies have shown that the exposure of cultured LSCs to an amniotic membrane (AM) maintained the constant expression of ΔNp63 and the undifferentiated phenotype (K3−, K14+) [28] and that, after prolonged passaging, cultured LSCs could be used in cell therapy for ocular surface repair [29]. AM has been proven to contain multiple components, including Laminin 5 and HC-HA/PTX3, and can be used as a niche for adult stem cells to amplify stem cell populations [30,31]. Commercial AM products (such as AmnioGraft) are already available for use as ocular transplantation tissue grafts to repair tissue damage caused by ocular surface inflammation [32]. However, human AM products are expensive because of the mandatorily required safety confirmation of the preparation process.

Technological advancements in genetic engineering have increased its potential clinical applications over the past decade (e.g., RNA vaccines, cancer vaccines) [33,34]. Cell proliferation can be boosted by manipulating gene regulation, and cultured LSCs have a potential use in cell therapy for the treatment of corneal disorders. Although STAT3/ΔNp63 interaction regulates the switch between CEC proliferation and differentiation, the direct genetic manipulation of *ΔNp63* may elevate the risk of carcinogenesis because of the high degree of homology between the DNA binding sites in *p63* and *p53*. The results of the present study demonstrate that the genetic manipulation of *ATF3*, a downstream regulator of ΔNp63, is a feasible alternative for boosting CEC proliferation in culture and to circumvent the possible cross-reactivity between *p63* and *p53*. In addition to the low risk of canceration, the current findings indicate that ATF3 activation promotes CEC proliferation but not differentiation. Therefore, larger numbers of CECs with proliferation potential may be generated by stimulating *ATF3* promoter activity. In summary, the current findings provide insights for the development of safe methods to boost CEC proliferation in culture for the purpose of cell therapy.

## 5. Conclusions

We identified a novel molecular mechanism (ΔNp63/ATF3/CDK pathway) underlying the regulation of CEC proliferation. ΔNp63 upregulates *ATF3* mRNA expression by increasing *ATF3* promoter activity, and ATF3 protein subsequently regulates the expression of cyclin D and p27 ^Kip1^, resulting in an increased proliferation of CECs. The genetic manipulation of *ATF3*, a downstream effector ofΔNp63, avoids the risk of cross-reactivity between *p63* and *p53*, which may represent an effective means to boost the expansion of CECs in vitro for cell therapy.

## Figures and Tables

**Table 1:** *ATF3* expression is upregulated in *ΔNp63*-overexpressing CECs.

Gene Symbol	Entrez Gene No.	Gene Description	Array (log_2_)
*ATF3*	467	Activating transcription factor 3	3.764
*JUN*	3725	Jun oncogene	1.621
*EGR1*	1958	Early growth response 1	1.391
*PAX6*	5080	Paired box protein 6	0.893
*CEBPB*	1051	CCAAT/enhancer-binding protein beta	0.820

## Data Availability

The data that support the findings of this study are available from the corresponding authors upon reasonable request.

## Supplementary Materials

The following supporting information can be downloaded at: https://www.mdpi.com/article/10.3390/jpm13040700/s1, Table S1: Primer list for reverse transcriptase PCR.
