# Supplementary material for: Transcription Factor ATF3 Participates in DeltaNp63-Mediated Proliferation of Corneal Epithelial Cells
Source: J Pers Med. 2023 Apr 21;13(4):700. doi: 10.3390/jpm13040700 (PMC10142479; doi:10.3390/jpm13040700)
Supplement: Supplementary file 1 [file jpm-13-00700-s001.zip › jpm-2251518-supplementary.pdf]

Supplementary data

Table S1. Primer list for reverse transcriptase PCR

Gene	Primer sequence	Accession no.	Product size (bp)
ATF3	F: CCTCTGCGCTGGAATCAGTC<br>R: TTCTTTCTCGTCGCCTCTTTTT	NM_001206486.2	111
JUN	F: TCCAAGTGCCGAAAAAGGAAG<br>R: CGAGTTCTGAGCTTTCAAGGT	NM_002228.4	78
EGR1	F: GGTCAGTGGCCTAGTGAGC<br>R: GTGCCGCTGAGTAAATGGGA	NM_001964.3	149
PAX6	F: TGGGCAGGTATTACGAGACTG<br>R: ACTCCCGCTTATACTGGGCTA	NM_001604.5	111
CEBPB	F: CTTCAGCCCGTACCTGGAG<br>R: GGAGAGGAAGTCGTGGTGC	NM_005194.4	136
CCNA1	F: TTTGGGGTCCAGGCAGGTT<br>R: AGGGTACATGATTGCGGGAAA	NM_003914.3	111
CCNB1	F: GCAGCAGGAGCTTTTTGCTT<br>R: CCAGGTGCTGCATAACTGGA	NM_031966.3	118
CCND1	F: GCCGAGAAGCTGTGCATCTA<br>R: GAAATCGTGCGGGGTCATTG	NM_053056.2	120
CCNE1	F: CAGCCCCATCATGCCGAG<br>R: GGTCACGTTTGCCTTCCTCT	NM_001238.3	118
CDKN1B (p27)	F: AGTGTCTAACGGGAGCCCTA<br>R: CCGGGTTAACTCTTCGTGGT	NM_004064.4	115
CDKN1A (p21)	F: GCTGCCGAAGTCAGTTCCTT<br>R: GCATGGGTTCTGACGGACAT	NM_000389.4	117
